# Sleep restriction prior to antigen exposure does not alter the T cell receptor repertoire but impairs germinal center formation during a T cell-dependent B cell response in murine spleen

**DOI:** 10.1016/j.bbih.2021.100312

**Published:** 2021-07-30

**Authors:** Cornelia Tune, Julia Hahn, Stella E. Autenrieth, Martin Meinhardt, Rene Pagel, Andrea Schampel, Lisa-Kristin Schierloh, Kathrin Kalies, Juergen Westermann

**Affiliations:** a Institute of Anatomy, University of Luebeck, Germany; b Department of Internal Medicine II, University of Tuebingen, Germany

**Keywords:** Sleep deprivation, Antigen presentation, T cell-dependent B cell response, Sheep red blood cells, Germinal center, Mouse, Spleen, BCZ, B cell zone, CCL, C–C motif ligand, CCR, C–C motif receptor, CD, cluster of differentiation, CIITA, class II major histocompatibility complex transactivator, CXCL, C-X-C motif ligand, FDR, false discovery rate, GC, germinal center, IgG, Immunglobulin G, IL, interleukin, IFN, interferon, MHC-II, major histocompatibility complex II, RP, red pulp, SD, standard deviation, SLO, secondary lymphoid organ, SRBC, sheep red blood cells, TCR, T cell receptor, TCR-R, T cell receptor repertoire, TCZ, T cell zone, Tfh, follicular T helper cells

## Abstract

It is well known that sleep promotes immune functions. In line with this, a variety of studies in animal models and humans have shown that sleep restriction following an antigen challenge dampens the immune response on several levels which leads to e.g. worsening of disease outcome and reduction of vaccination efficiency, respectively. However, the inverse scenario with sleep restriction preceding an antigen challenge is only investigated in a few animal models where it has been shown to reduce antigen uptake and presentation as well as pathogen clearance and survival rates. Here, we use injection of sheep red blood cells to investigate the yet unknown effect on a T cell-dependent B cell response in a well-established mouse model. We found that 6 ​h of sleep restriction prior to the antigen challenge does not impact the T cell reaction including the T cell receptor repertoire but dampens the development of germinal centers which correlates with reduced antigen-specific antibody titer indicating an impaired B cell response. These changes concerned a functionally more relevant level than those found in the same experimental model with the inverse scenario when sleep restriction followed the antigen challenge. Taken together, our findings showed that the outcome of the T cell-dependent B cell response is indeed impacted by sleep restriction prior to the antigen challenge which highlights the clinical significance of this scenario and the need for further investigations in humans, for example concerning the effect of sleep restriction preceding a vaccination.

## Highlights

• First study of sleep restriction prior to antigen challenge on adaptive response.
• T cell proliferation and T cell receptor repertoire remain unchanged.
• Germinal center development is dampened and antibody titer reduced.
• Effects stronger than if antigen challenge precedes sleep restriction in same model.

## 1 Introduction

It is a long known phenomenon of everyday life that sleep promotes protective functions of the immune system and by now well documented by a plethora of human and animal studies (reviewed e.g. by (Bryant et al., 2004) and (Besedovsky et al., 2019)). In healthy subjects, sleep has a regulatory effect on the host defense system, as it impacts tissue distribution and functionality of basically all types of immune cells (Benedict et al., 2007; Bonacho et al., 2001; Born et al., 1997; Dimitrov et al, 2007, 2009; Esquifino et al., 2004). The clinical relevance of sleep becomes evident by an association between sleep duration with e.g. pneumonia incidence (Patel et al., 2012), and sepsis outcome (Friese et al., 2009) as well as a sleep-induced improvement of vaccination efficiency (Benedict et al., 2012; Lange et al, 2003, 2011; Spiegel et al., 2002). While the majority of these studies determined quantitative blood parameters like lymphocyte numbers, cytokine levels and antibody titers in humans, we used mice to investigate sleep effects on the milieu of secondary lymphoid organs (SLO) and found its impact to be more pronounced in spleen than in lymph nodes (Tune et al., 2020). Accordingly, we determined the effects of sleep on the response to sheep red blood cells (SRBC), a blood born antigen processed in spleen. When 6 ​h of sleep restriction followed the antigen challenge we found synchronized reductions in the expression of genes reflecting antigen presentation (Tune et al., 2020). However, this did not have any significant long-term effects on the subsequent T cell-dependent B cell response, which prompted us to ask if the sleep induced changes in SLO milieu rather influence an upcoming immune response than an ongoing one. In light of the fact that sleep deprivation preceding an antigen exposure is as likely in daily life as is the other way round, only a surprising low number of studies investigated this scenario so far (Casey et al., 1974; Hahn et al., 2020; Lungato et al., 2015; Toth et al., 1995). Supporting our finding that sleep restriction reduces antigen presentation, Casey et al. found that uptake of the same antigen (SRBC) into the spleen was reduced after a period of 72 ​h of sleep deprivation. Likewise, Hahn et al. found the numbers of monocytes and thus antigen presenting cells in the spleen reduced by half after the same 6 ​h of sleep restriction. Pursuing the hypothesis of reduced antigen presentation, Hahn et al. challenged sleep restricted mice with *Yersinia enterocolitica* as a model of sepsis, while Lungato et al. infected sleep deprived mice with the murine malaria parasite *Plasmodium chabaudi*. Thus, both used a blood borne antigen and both reported a drastic decrease of survival rate in sleep deprived animals, which highlights the clinical relevance of this scenario. However, none of these studies investigated the adaptive component of the immune system that is induced by antigen presentation and likely contributed to the observed differences in pathogen clearance and survival. To explore whether sleep restriction prior to an antigen exposure also affects the adaptive immune response we here repeated our previous study (Tune et al., 2020) that combined 6 ​h of stress-free sleep restriction (Hahn et al., 2020; Husse et al., 2012) with SRBC injection which evokes a well described T cell-dependent B cell response (Stamm et al., 2013; Textor et al., 2018; Tune et al., 2020), but reversed the order of manipulations in such that sleep restriction preceded the antigen challenge. We searched for differences between sleep restricted mice and animals with undisturbed sleep in T cell proliferation and germinal center (GC) development, gene expression and the T cell receptor repertoire (TCR-R) as well as SRBC-specific antibody serum levels at the two most relevant time points, i.e. the peak of T cell proliferation 3 days (3 ​d) and the peak of B cell proliferation 10 ​d after SRBC injection (Stamm et al., 2013).

## 2 Material & methods

### 2.1 Mice

Animal experiments were performed in strict accordance with the German regulations of the Society for Laboratory Animal Science (GVSOLAS) and the European Health Law of the Federation of Laboratory Animal Science Associations (FELASA). The protocol was approved by the Regierungspraesidium Tuebingen (permit no M11/14). All efforts were made to minimize suffering of the animals. 12-week-old female C57BL/6 ​J mice were purchased from Janvier (St Berthevin Cedex, France), kept at constant temperature (20.0 ​± ​0.5 ​°C) and humidity (50–60%) and had ad-libitum access to standard food and water. The mice were group-housed (4–5 animals per cage) under a 12:12 ​h dark:light (50 lux) cycle.

### 2.2 Sleep restriction and immunization

Half of the mice (‘awake’ group) were kept awake for 6 ​h by gentle handling as described previously (Hahn et al., 2020; Husse et al., 2012; Tune et al., 2020), while the other half was left completely undisturbed during this time period (‘sleep’ group). Immediately after this sleep (restriction) phase, both mouse groups were immunized by injection of 10^9^ SRBC (Labor Dr. Merk, Ochsenhausen, Germany) in 200 ​μl PBS as described previously (Stamm et al., 2013). Subsequently, all mice were left undisturbed until sacrification 3 ​d (n ​= ​6 per group) and 10 ​d (n ​= ​10 per group) post injection (*p.i.)*, respectively.

### 2.3 Tissue isolation and blood processing

Mice were sacrificed by exposure to an overdose of inhaled carbon dioxide followed by total blood withdrawal 3 ​d or 10 ​d after SRBC injection. Spleens were snap frozen and stored at −80 ​°C until further analysis. Full blood was harvested by heart puncture and allowed to clot. Subsequently, serum was separated by centrifugation at 2000×*g* for 15min and stored at −80 ​°C until further processing.

### 2.4 ELISA for identification of SRBC-specific IgG antibodies

Flat bottom 96-well microtiter plates (Maxisorp 446612, Nunc) were coated with SRBC using a suspension of 1 ​× ​10^8^ SRBC in 0.05 ​ml PBS with overnight incubation at 4 ​°C. Subsequently, plates were washed and non-specific binding sites blocked with 1% skim milk in PBS for 1 ​h at room temperature. Individual sample sera of mice as well as a reference serum (RS, pooled sera of SRBC-immunized mice from previous experiment) and a normal mouse serum (NMS, pooled sera of naïve mice from previous experiments) were added to the wells and incubated for 1 ​h at room temperature. Thereafter, HRP-conjugated rabbit-anti-mouse IgG (H ​+ ​L; 1:500; 210-120-02, BioFX Laboratories) was added and incubated 1 ​h at room temperature in the dark, followed by addition of TMB substrate (Invitrogen) and incubation for 10–15 min. The color reaction was stopped by adding 2 ​M H_2_SO_4_ and detected at 405 ​nm using a microtiter plate reader. Relative IgG was calculated as quotient of optical density values (OD_sample_-OD_MNS_)/(OD_RS_-OD_NM__S_).

### 2.5 Histological analysis

Frozen spleens were cut into 12 ​μm thick cryosections and stained by immunohistochemistry using a monoclonal biotinylated antibody (B220 for B cells; BD Biosciences) to visualize B cell zones (BCZ) (Stamm et al., 2013). To visualize proliferating cells and thereby GC, we stained for Ki-67 (TEC-3; DakoCytomation) (Barthelmann et al., 2012). Digital images were taken using Axiophot Microscope and AxioCam (Carl Zeiss). Cell counts and GC area determination were performed with ImageJ (National Institutes of Health) as described previously (Tune et al., 2020).

### 2.6 RNA isolation, cDNA synthesis, and real-time RT-PCR

Five splenic cryosections (12 ​μm) per spleen were lysed in QIAzol lysis reagent and total RNA was extracted using the RNeasy® Plus Universal Mini Kit (Qiagen). RNA quantity was determined using the Quantus fluorometer (Promega Biosystems). Translation of 800 ​ng of total RNA into cDNA was performed using 200 ​ng of random primer, 0.01 ​M DTT, 1 ​μl reaction buffer, 0.5 ​mM dNTP (each obtained from Promega), and 100 U reverse transcriptase Superscript II RNase H Minus (Invitrogen Life Technologies) in a total volume of 20 ​μl. Samples were incubated at 42 ​°C for 50 min. Messenger RNA expression levels were determined by quantitative real-time PCR (qPCR) using the SDS ABI 7000 or SDS ABI 7900 system (Applied Biosystems). Relative abundances of target gene transcripts in a given sample were calculated as differences in cycle of threshold (CT) compared with the geomean expression of the four independent housekeeping genes *β-actin*, *gapdh*, *mln51* and *hprt* (ΔCT), and normalized to the ‘sleep’ group (ΔΔCT). Primer and probe sequences as well as gene accession numbers are provided upon request.

### 2.7 CDR3 sequence analysis of the TCRβ-chain

T cell receptor (TCR) β-chain transcripts were amplified from total RNA using a two-step reaction kit according to the manufacturer's protocol (iRepertoire; patent no. 7.999.092). Gene-specific primers targeting all V and J genes were used for reverse transcription and first-round PCR (OneStep RT-PCR Mix; Qiagen). During second-round PCR sequencing adaptors A and B for Illumina paired-end sequencing were added (Multiplex PCR Kit; Qiagen). The obtained PCR products were run on a 2% agarose gel and resulting 300 ​kb bands purified using QIAquick Gel Extraction Kit (Qiagen). Concentration of the extracted TCRβ libraries was determined and adjusted according to the PerfeCTa-NGS-Quantification Kit (Quantabio) and sequenced using the Illumina MiSeq Reagent Kit v2 300-cycle (150 paired-end read; Illumina), gaining an average of ≈1.8 ​× ​10^6^ reads per sample for the 3 ​d *p.i*. time point and ≈1.3 ​× ​10^6^ for the 10 ​d *p.i*. time point, without significant differences between the ‘sleep’ and awake’ group. Since we did not intend to compare 3 ​d with 10 ​d data, we decided against an adjustment of read counts by downsampling. CDR3β identification and correction of sequencing errors such as removal of nonfunctional CDR3β sequences were performed using ClonoCalc software (Fähnrich, 2017). We only considered sequences that were detected at least two times, and different nucleotide sequences that code identical amino acid sequences are treated as equal and are referred to as ‘clonotype’.

### 2.8 Statistics

Prism 7.0 (GraphPad Software Inc.) was used for layout and statistical testing of histological image, qPCR and ELISA data. Due to small sample size, partial absence of normal distribution and variance homogeneity as well as the fact that not every parameter was assessed at both time points, we decided on consequent testing for sleep effects and thus statistical differences within either the 3 ​d or 10 ​d time point only using the Mann-Whitney-U-Test. However, where applicable, we additionally performed two-factorial ANOVA with Sidak's post hoc testing. Correction for multiple comparisons was performed using Holm's method and correlation evaluated by calculation of Pearson's correlation coefficient r^2^.

Analysis of the CDR3β sequence was performed using the R platform for statistical computing (Inagaki Katashiba et al., 2019). Analog to our previous study (Tune et al., 2020) we characterized the TCRβ repertoire as a whole, as well as distinct fractions of clones defined by their copy number, e.g. the ‘top100’ fraction embracing the 100 clonotypes with the highest copy number. As intra-individual parameters we determined number of clonotypes, copies per clonotype, and the mean amino acid sequence length of the individual CDR3β repertoire, as well as usage of V and J segments. Concerning the latter, we focused on summation of those three segments detected most frequently in the top 100 fraction of naïve mice in our previous study (Tune et al., 2020), i.e. V12–2, V16, V19 and J1-1, J2-4, J2-7, respectively. Furthermore, we quantified the similarity of the repertoires of different mice as an inter-individual parameter by analyzing the clonal overlap determined by the Jaccard index. Due to multiple dependencies between the indices of each group the application of inferential statistics is problematic, which is why we restricted our analysis on descriptive considerations for this parameter.

## 3 Results

### 3.1 Sleep restriction prior to antigen exposure dampens GC development

**Fig. 1:**
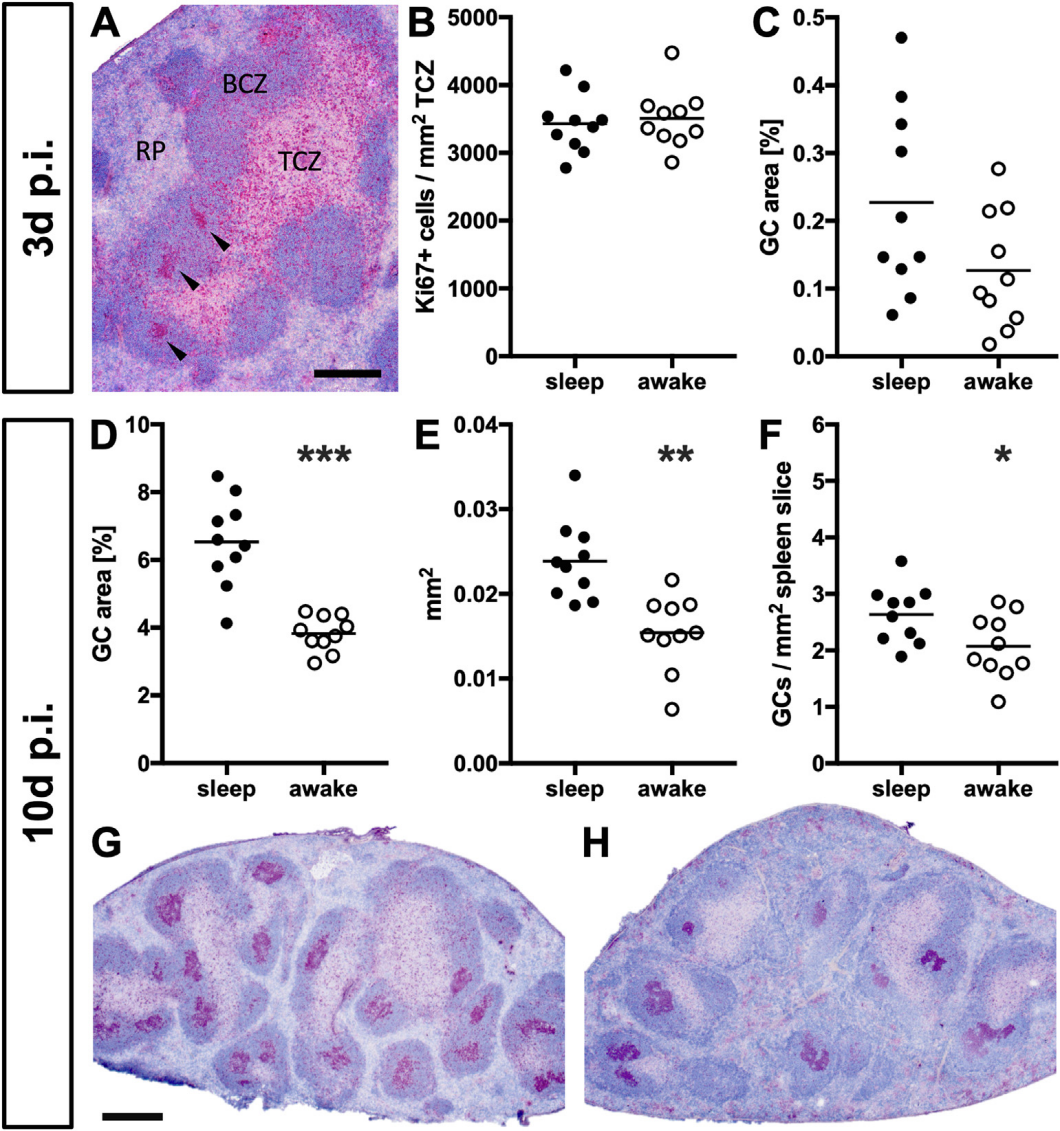
**Sleep restriction dampens GC development.** Naïve, 12-week old C57BL/6 mice were either allowed to sleep normally (‘sleep’) or subjected to 6 ​h of sleep restriction by gentle handling at the beginning of the sleeping phase (‘awake’). Directly thereafter both groups were immunized by injection of SRBC and investigated 3 ​d (n ​= ​6) and 10 ​d *p.i.* (n ​= ​10), respectively**. (A)** Representative spleen slice of a mouse with normal sleep at 3 ​d after immunization with SRBC; stained with the B-cell marker B220 (blue) visualizing the different spleen compartments: B cell zone (BCZ), T cell zone (TCZ), and the red pulp (RP). Staining with the proliferation marker Ki-67 (purple) reveals developing GC (arrowhead), i.e. cluster of proliferating cells within the BCZ. Bar equals 200 ​μm. Quantification of **(B)** proliferating cells within the TCZ at 3 ​d *p.i*, relative area of GC at **(C)** 3 ​d *p.i.* and **(D)** 10 ​d *p.i*., followed by the determination of GC **(E)** size and **(F)** number at 10 ​d *p.i*.; bar indicates mean, and p-values obtained by Mann-Whitney-U test followed by Holm's correction for multiple comparisons are displayed as ∗ p ​< ​0.05, ∗∗p ​< ​0.01, ∗∗∗p ​< ​0.001. Representative spleen slices of animals 10 ​d *p.i.* from the ‘sleep’ **(G)** and the ‘awake’ **(H)** group, respectively. Bar in (G) represents 500 ​μm and also accounts for (H). (For interpretation of the references to color in this figure legend, the reader is referred to the Web version of this article.)

We subjected mice to 6 ​h of sleep (‘sleep’ group) or wakefulness (‘awake’ group) during the first half of their regular resting phase, and directly thereafter immunized both groups with SRBC. After 3 ​d and 10 ​d, respectively, spleens were harvested and cell proliferation visualized via histochemical staining for Ki-67 and B220, which allows us to assess both T and B cell proliferation within the T cell zone (TCZ) and the developing GC, respectively (see Fig. 1A). We did not detect any differences in T cell proliferation either at 3 ​d (Fig. 1B) or 10 ​d *p.i.* (not shown) but found a reduction in GC total area with tendencies already at the beginning of GC development at 3 ​d (Fig. 1C, U ​= ​28, p ​= ​0.1051). This effect became significant during full GC development at 10 ​d *p.i.* (Fig. 1D, U ​= ​3, p ​< ​0.0001), and was validated by two-way ANOVA (interaction: F (1,18) ​= ​20.11, p ​= ​0.0001; time: F ​= ​295.9, p ​< ​0.0001, sleep: F ​= ​22.88, p ​< ​0.0001; sleep vs awake, 3 ​d: ns, 10 ​d: p ​< ​0.0001). Detailed analysis revealed that reduction in total area resulted from both fewer (Fig. 1E, U ​= ​12, p ​= ​0.0029) and smaller GC (Fig. 1F, U ​= ​21.5, p ​= ​0.0304). Thus, GC development was dampened by sleep restriction preceding the antigen challenge.

### 3.2 Sleep restriction prior to antigen exposure does not affect splenic gene expression during the T cell response

**Fig. 2:**
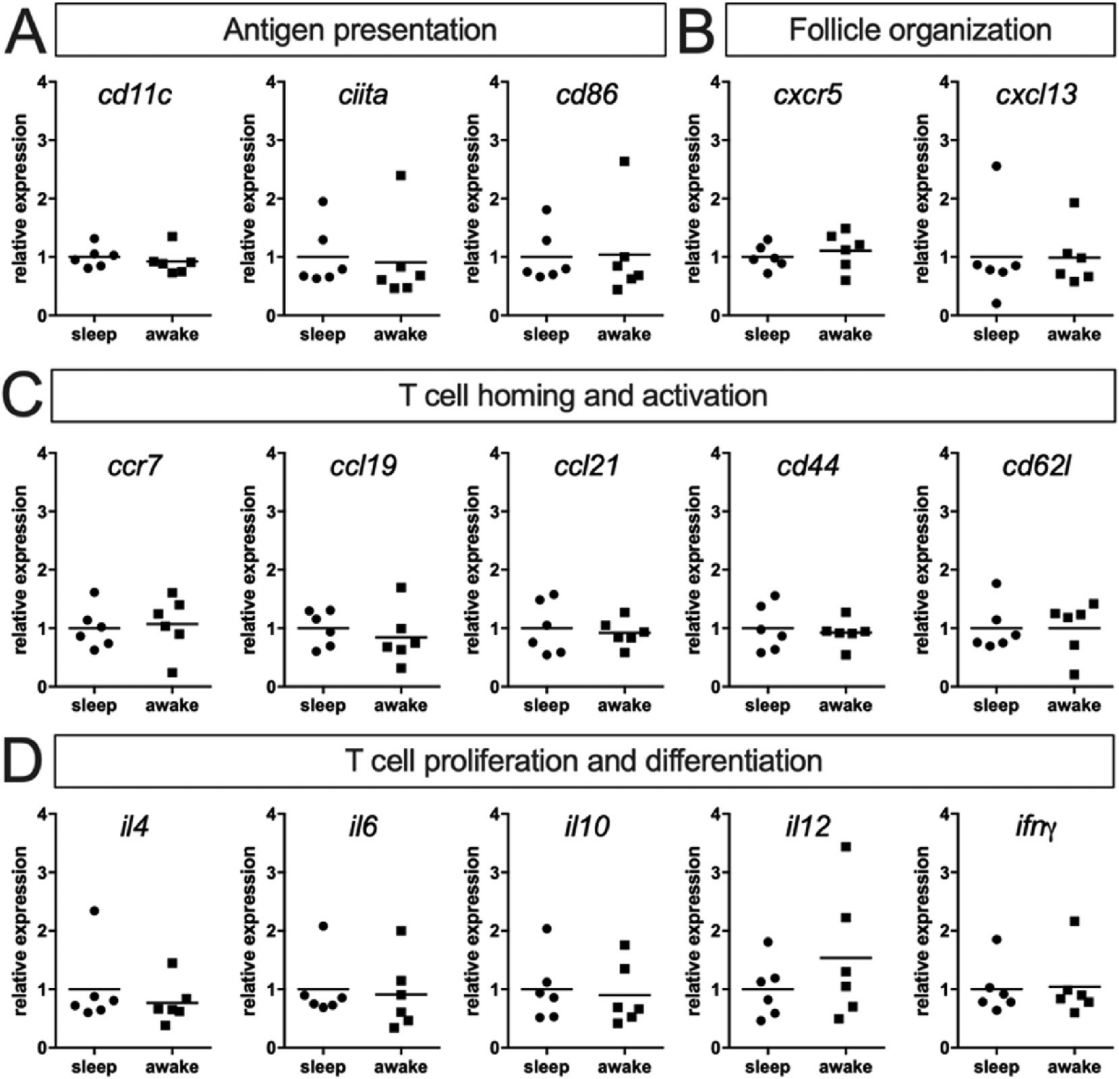
**Sleep restriction does not alter gene expression 3d after immunization.** Gene expression levels of the spleen taken 3 ​d after injection with SRBC were assessed by qPCR and normalized to the ‘sleep’ group (n ​= ​6 each). **(A**–**D)** Investigated genes included surface molecules (e.g. *cd11c*, *cd86*), chemokines (e.g. *cxcl13 and ccl19*) and their receptors (*cxcr5* and *ccr7*), as well as cytokines (e.g. *il4, il10 and ifnγ*) and are grouped according to their indicated function (for a detailed description of gene names and function see supplementary table 1). Statistical analyses using the Mann-Whitney-U-Test did not reveal any differences between the two groups, bars indicate mean values.

To assess if immune cells involved in a splenic T cell-dependent B cell response are functionally affected by sleep restriction, we determined expression levels of gene clusters reflecting important functions relevant for the respective time point and thus phase of the immune reaction. Corresponding to the histological data, at 3 ​d *p.i*. and thus within the T cell phase we did not find any differences in the expression of genes reflecting either antigen presentation (Fig. 2A), follicle organization (Fig. 2B), T cell homing and activation (Fig. 2C), or T cell proliferation and differentiation (Fig. 2D), respectively.

### 3.3 Sleep restriction prior to antigen exposure alters splenic gene expression during the B cell response

**Fig. 3:**
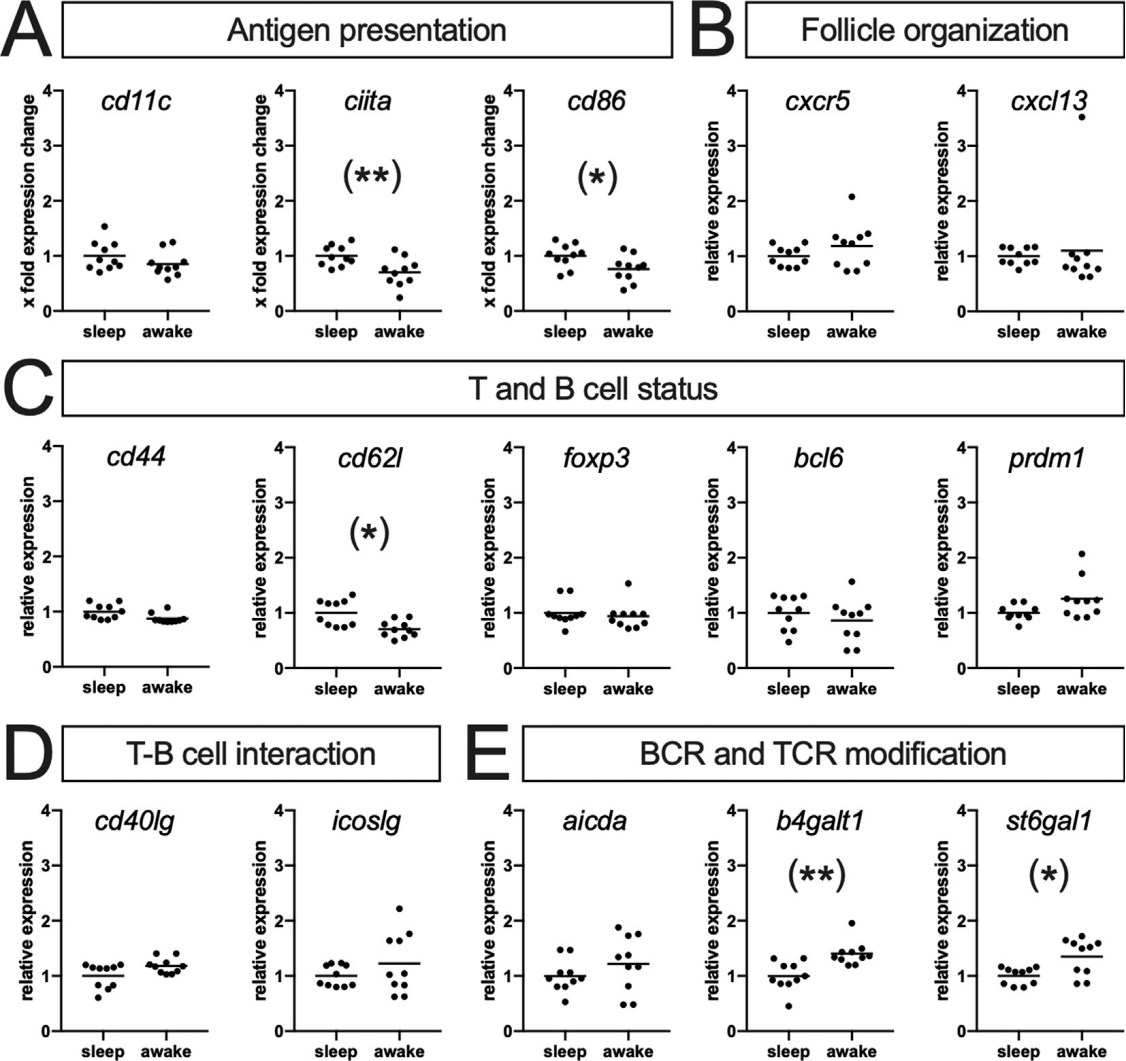
**Sleep restriction changes gene expression 10d after immunization.** Gene expression levels of the spleen taken 10 ​d after injection with SRBC were assessed by qPCR and normalized to the ‘sleep’ group (n ​= ​10 each). **(A**–**D)** Investigated genes included surface molecules (e.g. *cd11c*, *cd86*), transcription factors (e.g. *ciita*, *bcl6*), and enzymes (e.g. *b4galt1*, *st6gal1*) and are grouped according to their indicated function (for a detailed description of gene names and function see Supplementary table 1). Bar indicates mean value, and p-values obtained by Mann-Whitney-U test are displayed as ∗ p ​< ​0.05, ∗∗p ​< ​0.01. Parentheses indicate that none of the p-values reaches the significance level after correction for multiple testing using Holm's method.

Likewise corresponding to the histological finding of dampened GC development in sleep restricted animals at 10 ​d *p.i.*, we found the expression of genes reflecting antigen presentation slightly reduced at this time point which captures the B cell phase (Fig. 3A, *ciita*: U ​= ​16, p ​= ​0.0089; *cd86*: U ​= ​23, p ​= ​0.0433). On the other hand, genes encoding the chemokine system that recruits T and B cells into GC were not altered (Fig. 3B). Concerning the status of T cells, *cd44* as marker of effector T cells was unaltered, while *cd62l* as marker for central memory T cells was reduced (Fig. 3C, U ​= ​16, p ​= ​0.0089). *Foxp3*, which functions as marker for regulatory T cells was unaltered, as was *bcl6*, which induces the differentiation of naïve T helper cells into follicular T helper cells (Tfh). In B cells, Bcl6 suppresses the differentiation into antibody producing plasma cells and is thus an antagonist of Blimp1, a transcription factor encoded by *prdm1*. Similar to the genes we selected to capture interactions between T and B cells (Fig. 3D) as well as those involved in posttranslational modification of the T and B cell receptor, respectively (Fig. 3E), *prdm1* as indicator of antibody production showed a slight increase in gene expression (Fig. 3C). While this increase was a trend only (p ​< ​0.1) for *prdm1*, *cd40lg*, and *icoslg*, respectively, it reached significance for the two glycosylation enzymes *b4galt1* (U ​= ​6, p ​= ​0.0003) and *st6gal1* (U ​= ​21, p ​= ​0.0288).

However, none of the p-values obtained by single comparison reached the significance level after correction for multiple testing of all 15 genes. But genes reflecting similar functional aspects displayed changes in similar directions while gene groups of different functions showed expression changes in different directions, which leaves both effects obtained by chance and falsification of the results by e.g. expression changes of the reference genes unlikely. Given this and the fact that according to our previous study sleep dependent alterations in this model were expected to be minimal (Tune et al., 2020), we consider these observations as true effects and conclude that sleep restriction prior to an antigen challenge alters T and B cell gene expression, especially concerning antigen presentation, formation of central T memory cells, and glycosylation activity during the B cell phase.

### 3.4 Sleep restriction prior to antigen exposure does not alter features of the TCR repertoire

**Fig. 4:**
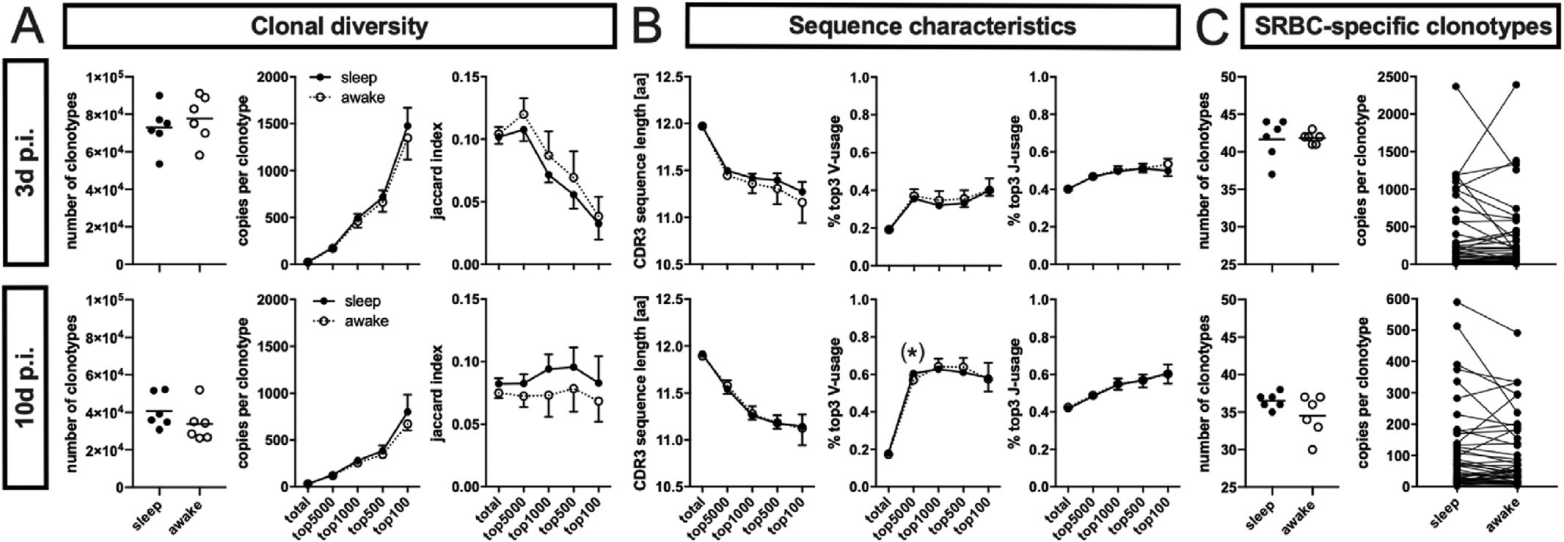
**Sleep restriction does not alter the TCR-R recruited intotheimmune response.** Deep sequencing analysis of the CDR3β region of the TCR-R of whole spleen cryosections at 3 ​d *p.i.* (upper panel) and 10 ​d *p.i.* (lower panel) of mice without (‘sleep’) and with sleep restriction (‘awake’) with n ​= ​6 each. Clonotypes grouped according to their copy number, total: all clonotypes displayed (left graph); top5000, top1000, top500, and top100: only the 5000, 1000, 500, 100 clonotypes with highest copy number, respectively. Data given as means with SD (n ​= ​6 per group) **(A)** Parameters of clonal diversity including the Jaccard index indicating clonal overlap (1 ​= ​100%; 0 ​= ​0%). **(B)** Parameters of sequence characteristics: CDR3β sequence length (number of amino acids, aa) and percentage of the three V and J segments most abundant in the top100 fraction of naïve mice, identified previously (V12–2, V16, V19, and J1-1, J2-4, J2-7, respectively; see Fig. S1 for single analysis of all V and J segments). (∗) indicates p ​< ​0.05 derived from single Mann-Whitney-U-Test that did not survive FDR correction. **(C)** Occurrence of 44 SRBC-specific clonotypes identified previously via differential gene expression analysis which selected sequences expanded compared to naïve and present in 75% of immunized animals and thus identified so-called public clones only. Left: number of SRBC-specific public clonotypes detected per mouse; right: mean copy number of each sequence within the respective group, lines connect same sequence to indicate average expression changes between sleep and awake group. All 44 sequences were detected at least in 2 mice per group (see Supplementary table 2 for details).

Since T cell proliferation was unchanged (Fig. 1), while gene expression analysis suggested a decrease in antigen expression (Fig. 3), the changes in B and T cell function might rather be of qualitative then quantitative nature. To address this possibility, we determined the TCR-R in whole spleen cyrosections by deep sequencing of the CDR3 region of the β−chain at both time points. Because we were primarily interested in the functional repertoire recruited into the evoked T cell-dependent B cell response, we merged sequences with different nucleotide sequences, i.e. different T cell clones, but identical amino acid sequences into a ‘clonotype’ with identical TCRs. Furthermore, besides characterizing the repertoire as a whole, we especially focused on the reacting, i.e. expanding clonotypes by separate analysis of those fractions with the most abundant clonotypes, e.g. the top100 and top500 fraction encompassing the 100 and 500 clonotypes with the highest copy number, respectively (Fig. 4). First, we assessed the diversity of the repertoire by determining number of clonotypes, copies per clonotype and the Jaccard index which measures the clonal overlap between animals expressed as percentage (Fig. 4A). The Jaccard index of about 10% in the total repertoire at 3 ​d *p.i.* (Fig. 4A, top right) means that 10% of all clonotypes are shared between two animals, while the gradual decrease of the Jaccard index throughout the top fractions indicates that the immune response against SRBC is (as shown in our previous studies (Textor et al., 2018; Tune et al., 2020) primarily of a so-called private nature, i.e. the expanding clonotypes in each animal are different and thus unique (Greiff et al., 2015; Venturi et al., 2006).

Next, we determined sequence characteristics (Fig. 4B), i.e. the length of the CDR3β region as well as the usage of V and J segments. These parameters reflect predictable distributions of clonotype features within the TCR-R based on the systematic differences between the likelihood of a clone being generated during somatic recombination and its probability to respond to an antigen. On the one hand, TCRs with few nucleotide additions are more likely generated than those with several nucleotide additions and are thus more abundant (Murugan et al., 2012), resulting in a reduction in mean sequence length throughout the top fractions (Fig. 3B, left). However, the probability of being specific for a certain antigen is on the other hand independent of sequence length which leads to an increase of mean sequence length within the top fractions during an immune response (Elhanati et al., 2014; Venturi et al., 2006). Similarly, some V and J genes are more abundant in naïve state and thus display a high proportion of usage within the top fractions (Fig. 4B, middle and right) that is reduced during an immune response due to expansion of antigen-specific clones with less frequently used V and J segments (Elhanati et al., 2014). We expected to see a slight disparity in these antigen-induced shifts within the TCR-R that we previously showed to occur also after immunization with SRBC (Textor et al., 2018; Tune et al., 2020), but did not find any relevant differences between animals with and without sleep restriction for any of the parameters and time points investigated. An initial significance within the top3 V usage of the top500 fraction (Fig. 4B; middle) did not survive FDR correction, nor did any of the few comparisons resulting in a significant difference in usage of a single V or J segment (see Supplementary Fig. S1). Furthermore, the latter were scattered throughout segments, fractions and time points, which is why we do not consider them as biological trends but statistical variations.

Finally, we assessed the occurrence of 44 clonotypes we identified as SRBC-specific in our previous study (Tune et al., 2020). To deduce antigen specificity of a TCR sequence from repertoire information only we selected sequences that were expanded in SRBC mice compared to naïve mice via differential gene expression analysis. Furthermore, clonotypes had to be present in at least 75% of immunized mice, meaning that the 44 sequences declared as SRBC-specific are so-called public clonotypes shared by most animals (Cibotti et al., 1994; Venturi et al., 2008). All 44 sequences could be found in the current dataset in at least 2 mice per group (see Supplementary Table 2), but neither the number of sequences detected per mouse nor their expression levels displayed significant or systematic changes between the ‘sleep’ and ‘awake’ group (Fig. 4C). Both these particular clonotypes (Fig. 4C) and total clonotypes (Fig. 4A) displayed a trend of reduction throughout the repertoire between ‘sleep’ and ‘awake’ groups at 10 ​d *p.i.* and between 3 ​d and 10 ​d *p.i.* in general in both number of clonotypes and their respective copy number and with the former also the Jaccard index. This, however, is due to technical variability of total read count between samples (see M&M) that we decided not to correct for since the differences did not reach significance between the two 10 ​d groups and no comparisons were conducted between 3 ​d and 10 ​d groups. Accordingly, we conclude that sleep prior to an antigen challenge does not result in detectable alterations of the TCR-R.

### 3.5 Sleep restriction prior to antigen exposure reduces SRBC-specific IgG serum level

**Fig. 5:**
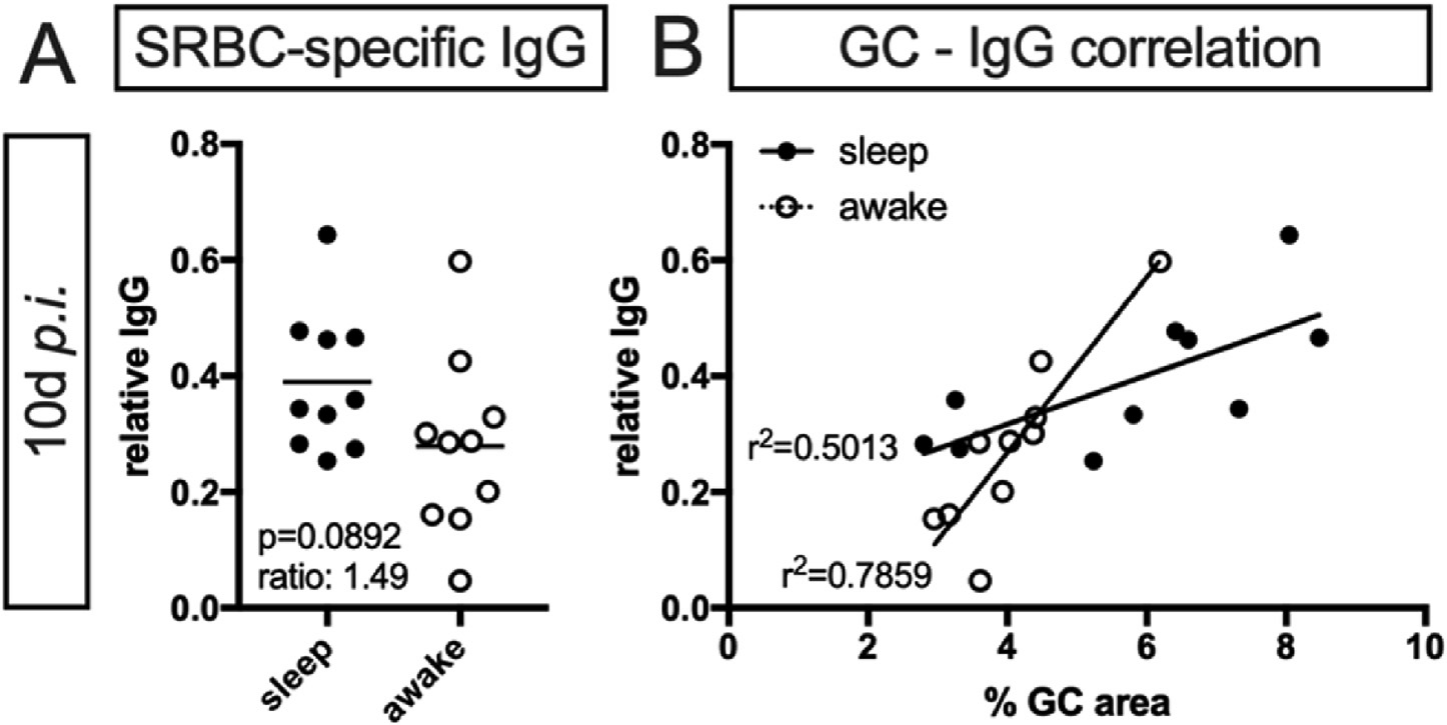
**Sleep restriction reduces SRBC-specific IgG serum levels in correlation with GC development. (A)** SRBC-specific IgG serum levels of n ​= ​10 animals each with normal sleep (‘sleep’) and animals subjected to sleep restriction (‘awake’) at 10 ​d *p.i.* assessed by ELISA; p-value given in graph derived from Mann-Whitney *U* test. **(B)** Correlation plot for SRBC-specific IgG levels in blood and relative GC area in spleen. Correlation assessed for each group by Pearson's correlation coefficient r^2^ given in graph.

The final outcome of a T cell-dependent B cell response and thus the clinically most important parameter is the level of antigen-specific antibodies. Accordingly, we determined serum levels of SRBC-specific immunoglobulin G (IgG) at 10 ​d *p.i.*, which we found on average reduced in animals subjected to sleep restriction (Fig. 5A). However, due to small number of samples paired with high variance the effect did not reach significance (U ​= ​27, p ​= ​0.0892). To assess if the observed reduction might rather be a statistical artefact or the expected minimal but true effect, we asked if IgG serum level and GC development, which we found to be significantly reduced in the ‘awake’ group (see section 3.1. and Fig. 1, respectively), correspond within each mouse. Indeed, we found a strong correlation between relative IgG serum level and relative GC area for both groups (Fig. 5B, sleep: r^2^ ​= ​0.5013, slope difference to zero F ​= ​8.042, p ​= ​0.02; and awake: r^2^ ​= ​0.7859, slope difference to zero F ​= ​29.37, p ​= ​0.0006). Thereby directly connecting the findings from the GC as site of origin of antibody producing plasma cells, we consider the observed reduction in mean IgG serum levels more likely to be a real effect and allow us to conclude that sleep restriction prior to an antigen challenge results in lower antigen-specific IgG serum levels.

## 4 Discussion

This study aimed to explore if sleep restriction impacts a T cell-dependent B cell response against an antigen encountered directly after the disturbed sleeping phase. While the effect of sleep (restriction) on an ongoing immune response is well investigated, e.g. in the context of vaccination studies (Benedict et al., 2012; Lange et al, 2003, 2011; Spiegel et al., 2002), the inverse scenario is – although as likely – only rarely taken heed of. The few available studies showed that antigen uptake, presentation and clearance are reduced after sleep restriction prior to the challenge with a blood born pathogen (Hahn et al., 2020; Lungato et al., 2015), which lead us to hypothesize that the adaptive immune response would be affected as well. Thus, we chose SRBC as blood born antigen that elicits a well described T cell-dependent B cell response (Stamm et al., 2013; Textor et al., 2018; Tune et al., 2020), but instead of imposing sleep restriction on an ongoing immune response as in our previous study (Tune et al., 2020), we imposed the antigen reaction on a splenic immune system disturbed by a recent phase of sleep restriction. We compared control mice with undisturbed sleep to mice subjected to 6 ​h of sleep restriction by gentle handling directly prior to SRBC injection and focused our analysis on the T cell proliferation phase that peaks around 3 ​d *p.i.* as well as the B cell proliferation phase with GC development that reaches its maximum at 10 ​d *p.i.* (Stamm et al., 2013).

### 4.1 The T cell response is not affected by sleep restriction prior to the antigen challenge

The T cell response is initiated via antigen presentation and cytokine expression by splenic dendritic cells and results in activation, expansion and differentiation of antigen-specific T cell clones in the TCZ. However, we found T cell proliferation within the TCZ determined by immunohistochemistry (Fig. 1B) unaffected by sleep restriction. Similarly, splenic gene expression (Fig. 2) of *cd11c* as marker for dendritic cells, the MHC-II transactivator (*ciita*) as indicator of antigen presentation (Xiao et al., 2015) and *cd86* expressed by B cells, dendritic cells, and macrophages as costimulatory ligand inducing T cell activation (Bhatia et al., 2006) remained as unchanged as the expression of (i) the chemokines *ccl19*, *ccl21* and their receptor *ccr7* that recruit T cells and dendritic cells into the TCZ (Baekkevold et al., 2001), (ii) the surface adhesion molecules *cd44* and *cd62l* that mediate T cell extravasation into SLOs (Gonda et al., 2005) and (iii) a set of cytokines (*il2, il4, il6, il10, il12* and *ifnγ*) that regulate T cell differentiation and are known to be altered by sleep (Besedovsky et al., 2019; Qi, 2016). The inability to see any effects of sleep restriction might be due to the fact that potential changes are too subtle to be detected by analyzing the whole spleen and the total T cell population, respectively. We addressed the latter by determining the TCR-R and taking a closer look at those fractions of clonotypes with high copy number to track T cell clones that responded to SRBC (Fig. 4). While we beautifully reproduced both the immunization induced shifts within several TCR parameters throughout these fractions as well as the occurrence of certain SRBC-specific public clonotypes described in our previous study (Tune et al., 2020), even this thorough analysis failed to elaborate any differences between sleep restricted (‘awake’) and control (‘sleep’) mice. This could be due to three possible reasons: First, 6 ​h of sleep restriction as used in the present study might be too short to result in measurable effects. However, sleep induced changes of SLO milieu described above were observed after similar amounts of sleep restriction, which suggests that e.g. the reduction of antigen presentation (Hahn et al., 2020) is compensated within the T cell response. One mechanism by which this might be accomplished in this model is, second, the ceiling effect: in order to induce a T cell-dependent B cell response a high dosage of SRBC has to be applied (Stamm et al., 2013) and the resulting huge amount of antigen available in the spleen might still be sufficient to induce a full T cell response despite possible dampening effects of sleep restriction. Third, effects might be restricted to certain T cell subtypes or memory cell formation and thus only display in later stages of the immune response, e.g. in the B cell response or second encounter reactions. The reduction in expression of *cd62l*, which also functions as a marker for central memory cells (Gerberick et al., 1997), at 10 ​d *p.i.* (Fig. 3C) might be a hint that this is indeed the case suggesting further investigations of the secondary immune response in future experiments.

### 4.2 The B cell response is likely dampened by sleep restriction prior to the antigen challenge

The B cell response is characterized by the formation of GC where activated antigen-specific B cells proliferate, undergo somatic hypermutation and interact with activated Tfh via antigen presentation and co-stimulation, a mechanism by which high affinity B cell clones were actively selected to differentiate into IgG producing plasma cells (Biram et al., 2019; Gitlin et al., 2014). Here, we found hints that GC formation is hindered already by the time of GC initiation 3 ​d after the SRBC injection (Fig. 1C). 10 ​d after immunization and thus during the peak of B cell proliferation we found GC to be reduced in both size and number in sleep restricted mice (Fig. 1E–F), which correlated strongly with a decrease in SRBC-specific IgG serum levels (Fig. 4). Thus, sleep restriction preceding the antigen challenge dampened the B cell response. This might be due to a reduced antigen presentation by B cells toward Tfh, as gene expression of *ciita*, the master regulator of MHC-II, and *cd86*, an important costimulatory molecule, was slightly reduced at the 10 ​d time point (Fig. 3A). Furthermore, the small increase in expression of the two glycosylation enzymes *b4galt1* and *st6gal1* (Fig. 3E) gives a hint that besides antibody amount also antibody affinity, which is not only defined by paratope sequence but likewise glycosylation (Jennewein and Alter, 2017; van de Bovenkamp et al., 2018), might be affected. However, future studies targeting antibody glycosylation status and the B cell receptor repertoire are required to determine qualitative changes of the B cell response and thus the efficiency of the humoral defense system. Furthermore, investigation of the secondary immune response is needed to reveal if B cell memory is affected, and the mechanism via which sleep restriction prior to the antigen exposure impedes the B cell response remains to be elucidated. Concerning the latter, we found both genes known to be predominantly expressed by Tfh (*cxcr5 and bcl6*) as well as molecules reflecting B cell interaction with Tfh (*icoslg* and *cd40lg*) slightly enhanced at 10 ​d *p.i.* (Fig. 3C–D). This suggest that the processes taking place within the GC, especially stimulatory signaling between Tfh and B cells that lead to the selection of high affinity B cell clones (Biram et al., 2019; Gitlin et al., 2014) might be altered by sleep restriction. Accordingly, specific investigations on Tfh function and GC dynamics via separation of single cell types on the one hand and spleen compartments on the other are required to reveal if and how sleep restriction prior to an antigen challenge selectively influences certain components of the T cell-dependent B cell response.

### 4.3 The impact of sleep restriction might be greater on an upcoming antigen challenge than an ongoing one

An interesting aspect of the present study is its analogy to the previous one with both using the same immune response model (SRBC) and the same sleep restriction protocol (6 ​h of gentle handling at the beginning of the resting phase) leaving the reversed order of the two manipulations the only difference. In the previous study where antigen challenge preceded sleep restriction, we found subtle changes in spleen gene expression 3 ​d after the antigen challenge indicating a dampening of antigen presentation, but neither the T cell response (proliferation, TCR-R) nor the B cell response (GC development, IgG serum level) were affected. In the present study where sleep restriction preceded the antigen challenge, the B cell response and thus one of the major components of pathogen defense and memory formation seems to be dampened. Hence, we consider it likely that sleep restriction impacts an upcoming immune response on a functionally relevant level, while the impact on an ongoing one seems to be compensated during the course of the immune response (Tune et al., 2020). The latter was partially also the case in human vaccination studies (Benedict et al., 2012), while in those that found longer lasting effects (Lange et al, 2003, 2011; Spiegel et al., 2002) sleep restriction was longer compared to our model and/or applied repeatedly, which gives rise to the speculation that the impact might have been even stronger if sleep restriction preceded vaccination. This should be elucidated in future studies – preferably via experiments investigating both scenarios in parallel.

### 4.4 Further studies are required to evaluate true extent and relevance of the findings

Since this study was designed as explorative the number of animals was kept small which resulted in inconclusive p-values between 0.05 and 0.1 for several parameters. Thus, a repetition with considerably larger group size is required to reveal which of the found trends reveal as true effects, and more detailed parameters like the separation of T cell subtypes mentioned above need to be assessed to elucidate underlying cellular and molecular mechanisms by which sleep (restriction) exerts its effect on the immune response. Furthermore, to avoid possible interference of stress either due to dominance behavior or social isolation well described for the C57Bl/6 strain (Horii et al., 2017; Heck et al., 2020) male mice were not included, and stage of estrous cycle was not determined. Future studies should also address these sex-dependent sources of effect variability.

### 4.5 Conclusion

We showed here that sleep restriction prior to an antigen challenge likely dampens the B cell response and thus antibody production. This finding might be less relevant for infections with replicating pathogens where antigen presentation continues and newly activated T and B cells can join the ongoing immune reaction (Shulman et al., 2013) and thereby compensate for initial dampening effects of short term sleep restriction. However, symptoms might be worsened and disease duration prolonged due to the impaired initiation of the immune response, especially if sleep restriction exceeds the few hours used in the current study. Furthermore, our results suggest that even a single night of partial sleep restriction might reduce vaccination efficiency and that this effect is stronger if sleep is reduced in the night before the vaccination compared to the night thereafter, a scenario that to our knowledge has not been investigated yet. Hence, further studies in both humans and animal models are required to fully elucidate the clinical relevance and the mechanism by which sleep restriction dampens the T cell-dependent B cell response against an antigen encountered after a disturbed night.

## Author's contribution

CT conducted histological and qPCR experiments, assembled the figures and wrote the manuscript, KK, JH and SEA planned and conducted sleep experiments, MM and RP conducted the NGS analyses, LKS and AS gave critical advice, discussed the data and revised the manuscript, and JW organized experiments, gave critical advice and revised the manuscript. All authors read and approved the final manuscript.

## Declaration of competing interest

The authors declare no commercial or financial conflict of interest.
