# Supplementary material for: Sleep restriction prior to antigen exposure does not alter the T cell receptor repertoire but impairs germinal center formation during a T cell-dependent B cell response in murine spleen
Source: Brain Behav Immun Health. 2021 Jul 30;16:100312. doi: 10.1016/j.bbih.2021.100312 (PMC8474616; doi:10.1016/j.bbih.2021.100312)
Supplement: Multimedia component 1 [file mmc1.pdf]

Abbrev.	Full and alternative names	Relevant gene and marker function
aicda	Activation induced cytidine deaminase; Single-stranded DNA cytosine deaminase	DNA-mutating enzyme; expressed by B-cells during GC development; master regulator of somatic hypermutation and class switch recombination; inhibited by prdm1/blimp1 (see below: prdm1)
b4galt1	β-1,4-galactosyltransferase 1	Glycosylation enzyme; expressed ubiquitously;<br>Second to last enzyme of N-glykan synthesis preceding St6gal1 (see below)
bcl6	B cell lymphoma 6 protein	Transcription factor; expressed by T and B cells, master regulator of differentiation of naïve T helper cells into Tfh cells; inhibits differentiation of B cells into plasma cells during GC development; downregulated by CD40 and pdrm1 (see below)
ccl19	C-C motif chemokine ligand 19; Macrophage inflammatory protein-3-beta (MIP-3b)	Chemoattractant; expressed by reticular cells; recruits immune cells, especially naïve T cells, into SLOs (homing) and their trafficking into follicles
ccl21	C-C motif chemokine ligand 21	Chemoattractant; expression and function similar to ccl19
ccr7	C-C motif chemokine receptor 7	Receptor for ccl19 and ccl21; expressed by many immune cells including B and T cells; regulates lymphocyte homing and trafficking; stimulates dendritic cell maturation
cd11c	Cluster of differentiation 11c;<br>Integrin α-X	Surface protein; selectively expressed by monocytes and dendritic cells
cd40lg	Cluster of differentiation 40 ligand, CD154	Co-stimulatory molecule; expressed by activated T cells; activation of receptor CD40 expressed by B cells promotes plasma cell differentiation and function
cd44	Cluster of differentiation 44;<br>Homing cell adhesion molecule;<br>Lymphocyte homing receptor;<br>CD195	Cell adhesion molecule; expressed by most cell including immune cells; involved in cell–cell interactions, cell adhesion and migration; contributes to lymphocyte activation, recirculation and homing; strongly expressed by and therefor marker for effector-memory T-cells
cd62l	Cluster of differentiation 62L;<br>L-selectin	Cell adhesion molecule; expressed by leukocytes; functions as homing receptor by facilitating entry into SLOs; expressed by naïve T and B cells and central memory T cells; not expressed by effector memory T cells and plasma cells
cd86	Cluster of differentiation 86;<br>B7-2	Co-stimulatory molecule; expressed by antigen presenting cells; interaction with receptor CD28 expressed by T cells required for activation
ciita	Class II MHC transactivator	Transcription factor; master regulator of MHC-II genes
cxcl13	C-X-C motif chemokine ligand 13;<br>B lymphocyte chemoattractant;	Chemoattractant; expressed by follicular dendritic cells and T cells derived from the GC; recruits B cells into the follicle
cxcr5	C-X-C motif chemokine receptor 5;<br>Burkitt lymphoma receptor 1	Receptor for Cxcl13; expressed by B and T cells; essential role for migration of lymphocytes into follicles; marker molecule for Tfh cells
foxp3	Forkhead-box-protein P3	Transcription factor; expressed by regulatory T cells
il4	Interleukin 4	Cytokine; expressed by T helper cells type 2 (Th2), elevated 3d after SRBC injection
il6	Interleukin 6	Cytokine, expressed by antigen presenting cells, supports T and B cell proliferation and differentiation, suppresses function of regulatory T cells
il10	Interleukin 10	Cytokine; expressed by antigen presenting cells and lymphocyte subsets; supports Th2 differentiation and function; elevated 3d after SRBC injection
il12	Interleukin 12	Cytokine; expressed by antigen presenting cells after antigen contact; promotes T cell differentiation into T helper cells type 1 (Th1)
icoslg	Inducible T cell costimulatory ligand; CD275; B7H2	Co-stimulatory molecule; expressed by B cells; activation of receptor icos expressed by T cells promotes proliferation and cytokine secretion; autocrine secretion leads to proliferation and differentiation into plasma cells
ifnγ	Interferon gamma	Cytokine; expressed by T cells; activation of CD8+ cytotoxic T cells; induction of MHC-II expression by antigen presenting cells
prdm1	PR domain zinc finger protein 1;<br>B-lymphocyte induced maturation protein 1 *(blimp1)*	Transcription factor; expressed by most immune cells, including B and T cells; inhibits egress of T cells; leads to proliferation and differentiation of plasma cells; Bcl6 antagonist
st6galt1	ST6 β-galactoside α-2,6-sialyltransferase 1	Glycosylation enzyme; expressed ubiquitously; involved in regulation of T and B cell migration and Interaction via posttranslational modification of surface molecules including T and B cell receptors, especially relevant for IgG-isotype: st6galt1-mediated glycosylation determines pro- or anti-inflammatory function

Supplementary Table 1: Full gene names and their function. Note that several of these genes have a variety of functions of which only those relevant for the present study are given here. GC, germinal center; MHC, major histocompatibility complex; Tfh, follicular T helper cell

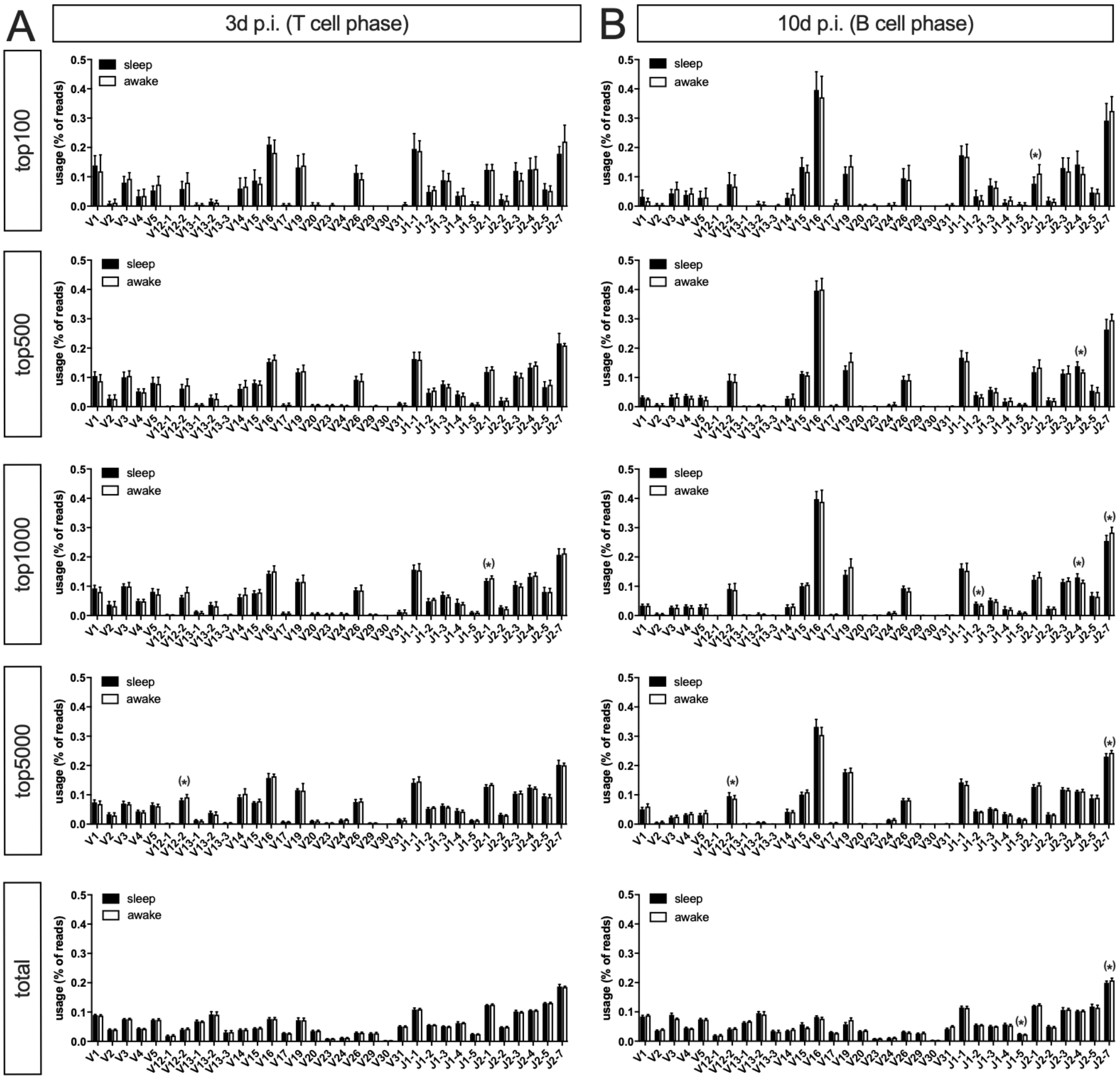

Supplementary Figure S1: Sleep restriction does not alter V and J segment usage within the CDR3β region of the TCR. Analysis of V and J segment usage after deep sequencing the CDR3β region of the TCR of whole spleen cryosections at **(A)** 3d *p.i.* and **(B)** 10d *p.i.* of mice without ('sleep') and with sleep restriction ('awake"'). Clonotypes grouped according to their copy number, total: all clonotypes detected; top5000, top1000, top500, and top100: only the 5000, 1000, 500, 100 clonotypes with highest copy number, respectively. Data given as means with SD (n=6 per group); (*) Indicates p-values <0.05 in single comparison via Mann-Whitney-U-Test that did not survive FDR correction.

Amino acid sequence	number of animals per sequence				average copy number per sequence			
	3d		10d		3d		10d	
	sleep	awake	sleep	awake	sleep	awake	sleep	awake
ASSLADSGNTLY	6	6	6	5	1188,5	1331,3	512,6	236,7
ASSLRGPYEQY	6	6	6	5	1012,7	1380,7	138,5	293,8
ASSLGDSGNTLY	6	6	6	6	1152,2	2389,7	589	491,2
ASGDRGDTEVF	6	6	6	6	194,5	215,2	28,5	53,8
ASSLRGGYEQY	6	6	6	6	1100,8	741,5	282,2	332,8
ASSLRSSYEQY	6	6	6	6	2367,8	1256,3	178,3	200,7
ASSLEGNSGNTLY	6	5	6	6	567,0	582,7	390,0	295,8
ASGDRGYAEQF	6	6	6	6	246,5	307,2	17,1	37,0
ASSSWGGYEQY	5	6	5	3	27,3	34,2	34,6	48,2
ASSLGGGQNTLY	6	6	6	6	287,7	439,7	336,0	154,7
ASSQGGYAEQF	4	5	2	3	24,7	17,3	37,0	56,2
TCSADLGGYAEQF	6	6	3	6	280,7	396,3	66,5	69,3
ASSLGGVQDTQY	6	6	5	3	30,2	35,8	55,6	15,0
ASGDARDTQY	5	6	5	5	163,0	31,2	31,5	7,7
ASSPGSSYEQY	6	6	6	6	177,0	451,3	97,5	187,3
ASGDRGGYEQY	5	4	4	6	39,3	54,2	19,0	12,2
ASSQDLGSSYEQY	6	6	6	4	98,2	72,7	35,3	36,5
ASSRLGGNTGQLY	5	6	4	3	15,2	62,3	13,6	11,5
ASSLSGSGNTLY	6	6	4	4	132,3	91,3	10,0	8,3
ASGDAGTGGYEQY	6	6	6	6	19,2	149,8	17,1	32,2
ASSPTGGETLY	5	4	5	3	63,3	68,2	67,8	33,2
AWSLGTGGYAEQF	5	4	2	2	21,3	8,0	3,3	4,5
ASSRQANTEVF	6	6	6	6	601,8	195,5	132,8	138,3
ASSLGVSGNTLY	6	6	4	6	1198,8	165,2	170,0	180,3
AWSLGGSYNSPLY	6	6	4	4	94,7	72,0	15,2	12,8
GARDNSGNTLY	6	6	5	6	48,0	103,8	21,0	17,0
ASSQGSGNTLY	6	6	6	5	924,3	329,3	87,1	50,7
ASGDWGQDTQY	6	6	5	6	24,3	14,3	10,0	18,2
ASSRDWGKDTQY	6	5	3	3	48,2	14,0	24,3	27,8
ASSQDRSQNTLY	6	5	4	4	145,3	147,5	80,0	38,3
ASSQDWGSSYEQY	6	6	6	6	399,7	239,3	107,8	87,8
ASSLGGSYEQY	6	6	6	6	724,0	603,5	374,3	333,8
ASSTGSSYEQY	5	6	4	4	50,7	85,5	71,8	84,0
TCSGDWGYEQY	5	6	6	4	991,7	638,0	118,5	73,3
ASSSGNTLY	6	6	6	5	162,5	190,3	182,8	132,5
ASSPTVSNERLF	6	6	5	4	108,3	102,2	26,1	31,5
ASSLRGGSDYT	5	6	5	5	17,7	44,5	45,5	28,2
ASSQDRGSSYEQY	4	5	4	5	71,2	17,7	17,5	51,5
ASSLVGNTEVF	6	5	6	2	226,8	57,5	71,6	46,8
ASSLTGGAREQY	6	6	6	4	223,7	221,0	127,3	101,2
ASSLTGDTEVF	5	5	4	5	58,5	32,3	9,1	69,2
ASSPLGSAETLY	6	6	3	3	16,3	128,0	27,0	16,7
ASSLDNSQNTLY	6	6	6	6	232,8	205,8	230,5	194,3
ASGDRGGNTLY	6	6	4	2	33,5	36,7	4,6	15,8

Supplementary Table 2: SRBC-specific public clones. Occurrence and mean copy number of 44 SRBC-specific clonotypes identified previously via differential gene expression analysis which selected sequences expanded compared to naïve and present in 75% of immunized animals and thus identified so-called public clones only. Total number of animals per group is 6; each sequence was detected in at least 2 mice per group; copy number given as mean per group calculated with copy number = 0 if sequence was not detected in an animal.
